# Association of Telomere Length with Breast Cancer Prognostic Factors

**DOI:** 10.1371/journal.pone.0161903

**Published:** 2016-08-29

**Authors:** Kaoutar Ennour-Idrissi, Bernard Têtu, Elizabeth Maunsell, Brigitte Poirier, Alicia Montoni, Patrick J. Rochette, Caroline Diorio

**Affiliations:** 1 Axe Oncologie, Centre de Recherche du CHU de Québec-Université Laval, Quebec city (QC), Canada; 2 Centre de Recherche sur le Cancer, Université Laval, Quebec city (QC), Canada; 3 Département de médecine sociale et préventive, Faculté de médecine, Université Laval, Quebec city (QC), Canada; 4 Département de biologie moléculaire, biochimie médicale et pathologie, Faculté de médecine, Université Laval, Quebec city (QC), Canada; 5 Centre des Maladies du Sein Deschênes-Fabia, Hôpital du Saint-Sacrement, Quebec city (QC), Canada; 6 Department de chirurgie, Faculté de médecine, Université Laval, Quebec city (QC), Canada; 7 Axe Médecine Régénératrice, Centre de recherche du CHU de Québec-Université Laval, Quebec city (QC), Canada; University of South Alabama Mitchell Cancer Institute, UNITED STATES

## Abstract

**Introduction:**

Telomere length, a marker of cell aging, seems to be affected by the same factors thought to be associated with breast cancer prognosis.

**Objective:**

To examine associations of peripheral blood cell-measured telomere length with traditional and potential prognostic factors in breast cancer patients.

**Methods:**

We conducted a cross-sectional analysis of data collected before surgery from 162 breast cancer patients recruited consecutively between 01/2011 and 05/2012, at a breast cancer reference center. Data on the main lifestyle factors (smoking, alcohol consumption, physical activity) were collected using standardized questionnaires. Anthropometric factors were measured. Tumor biological characteristics were extracted from pathology reports. Telomere length was measured using a highly reproducible quantitative PCR method in peripheral white blood cells. Spearman partial rank-order correlations and multivariate general linear models were used to evaluate relationships between telomere length and prognostic factors.

**Results:**

Telomere length was positively associated with total physical activity (r_s_ = 0.17, *P = 0*.*033*; *P*_*trend*_ *= 0*.*069*), occupational physical activity (r_s_ = 0.15, *P = 0*.*054; P*_*trend*_ *= 0*.*054*) and transportation-related physical activity (r_s_ = 0.19, *P = 0*.*019*; *P = 0*.*005*). Among post-menopausal women, telomere length remained positively associated with total physical activity (r_s_ = 0.27, *P = 0*.*016; P*_*trend*_ *= 0*.*054*) and occupational physical activity (r_s_ = 0.26, *P = 0*.*021*; *P*_*trend*_ *= 0*.*056*) and was only associated with transportation-related physical activity among pre-menopausal women (r_s_ = 0.27, *P = 0*.*015*; *P = 0*.*004*). No association was observed between telomere length and recreational or household activities, other lifestyle factors or traditional prognostic factors.

**Conclusions:**

Telomeres are longer in more active breast cancer patients. Since white blood cells are involved in anticancer immune responses, these findings suggest that even regular low-intensity physical activity, such as that related to transportation or occupation, could be recommended to breast cancer patients.

## Introduction

Breast cancer is the most common cancer in women worldwide, and the second most common cause of cancer death among women [1, 2]. According to the World Health Organization statistics [1, 2], between 2008 and 2012, incidence increased by more than 20% and mortality by 14%. In western countries, 5-year survival is around 89%, due to early detection and molecularly targeted therapies. However, traditional prognostic factors are still imprecise in predicting breast cancer prognosis and therefore new independent prognostic markers are needed.

Telomeres are highly specialized structures capping the ends of linear chromosomes [3–6]. They consist of repeated DNA sequences, 5’-TTAGGG-3’ of 5 to 15 kb length in humans, bound by multiple telomeric interacting proteins. Telomeres ensure the stability of chromosomes and genome integrity during replication. The telomerase enzyme complex, a specialized reverse transcriptase, extends the 3’ end of chromosomes by adding TTAGGG repeats [4, 6]. In absence of telomerase, gradual shortening of telomeres occurs with each cell division due to the end-replication problem (the inability of DNA polymerase to fully replicate chromosomes ends). When telomere shortening reaches a critical point, DNA damage responses are elicited, leading to replicative senescence and cell apoptosis (programmed cell death). Inflammation and oxidative stress have been shown to result in accelerated telomere shortening and several studies suggest that some lifestyle factors like smoking, alcohol abuse, sedentary lifestyle and obesity have an impact on telomere length in healthy individuals [7–10]. These modifiable factors also appear to be associated with breast cancer prognosis [11–13].

In cells that express telomerase, such as blood leukocytes, telomere length seems to be a dynamic feature that responds to processes that can shorten or lengthen telomeres [14]. Therefore, peripheral blood cell telomere length could be a surrogate for both the ability of underlying dynamic processes to restore or maintain telomere homeostasis and for assessing the impact of modifiable environmental factors. The systematic review of literature suggest a trend toward a positive association of longer telomeres with better prognosis [15]. However, the exact prognostic significance of telomere length for breast cancer patients is unclear.

The objective of the present study is to evaluate the association of telomere length, measured in peripheral blood cells, with traditional and potential prognostic factors in breast cancer patients.

## Materials and Methods

### Study design and population

We conducted a cross-sectional study on data collected at the time of surgery. Selection of study population was described elsewhere [16]. Briefly, 164 consecutive women who underwent surgery for unilateral breast cancer were prospectively recruited between January 2011 and May 2012, at a breast cancer reference center, the "Centre des maladies du sein Deschênes-Fabia du CHU de Québec" in Quebec City, Canada. Women were eligible (n = 226) if they were not older than 70 years, were not pregnant, had no previous diagnosis of cancer other than non-melanoma skin cancer, never had any breast surgery including breast reduction or implants, never took a selective estrogen receptor modulator such as Tamoxifen or Raloxifen, and did not receive any treatment prior to surgery. Of the consecutively approached women, 226 were eligible and 164 (73%) accepted to participate. Blood samples were provided by 162 participants. All participants provided written informed consent. The study protocol was reviewed and approved by the Research ethics committee of the Centre de Recherche du CHU de Québec.

### Data collection

Before surgery, a qualified research nurse performed anthropometric measures (weight and height measures) and drew blood samples according to standardized protocols. Information about risk factors was collected on average 24 days after surgery, using standardized questionnaires administered by telephone interview. Interviews included questions on gynecological and obstetric history, hormone use, and important lifestyle factors (smoking, alcohol consumption, physical activity). Questions on physical activity were derived from the Past Year Total Physical Activity Questionnaire [17], which measures all types (i.e., occupational, household, transportation-related and recreational) and all parameters (i.e., frequency, duration, and intensity) of physical activity and enables computation of physical activity data as metabolic equivalent (MET) hours of activity per week [17]. Biological characteristics of the tumor were extracted from pathology reports, including tumor size, lymph node involvement, histologic type, tumor grade, hormonal and growth factor receptor status. Disease stage was established following the American Joint Committee on Cancer (AJCC) cancer staging system for breast cancer [18].

### Telomere length measurement

Blood samples drawn before surgery were collected in EDTA-treated tubes and processed within 2 hours to obtain buffy coats, which were stored at −80°C until analysis. A salting-out method was used to extract DNA from 50 μl of buffy coat with Gentra PureGene Cell Kit (QIAGEN Inc., Canada) according to the manufacturer’s protocol for 3.5 x 10^6^ white cells. DNA quality and quantity were assessed using the NanoDrop® 2000c spectrophotometer (Thermo Scientific, Fisher Scientific Canada).

Mean relative telomere length was measured with the quantitative polymerase chain reaction (qPCR) method first described by Cawthon RM [19], with slight modifications. Briefly, the telomeric repeats (T) were amplified using primers that hybridize to telomeres but have mismatches across their length that prevent primer-dimers formation (i.e. hybridization of two primers); amplification was measured quantitatively and compared to that of a single copy gene (S), to adjust for the amount of DNA in the reaction, assuming that both products are amplified with similar efficiency. The result is a relative telomere length estimation, the T/S ratio. The sequences of telomere primers used were: 5’- GGT TTT TGA GGG TGA GGG TGA GGG TGA GGG TGA GGG T-3’ (forward) and 5’- TCC CGA CTA TCC CTA TCC CTA TCC CTA TCC CTA TCC CTA -3’ (reverse). The single copy gene human beta‐globin (Hbg) primers were: 5’- GCT TCT GAC ACA ACT GTG TTC ACT AGC -3’ (forward) and 5’- CAC CAA CTT CAT CCA CGT TCA CC -3’ (reverse). For each sample, 20 μl of reaction solution was prepared using 1 ng of genomic DNA diluted to 0.2 ng/μl, 10 μl of 2× Brilliant III Ultra-Fast SYBR® Green QPCR Master Mix (Agilent Technologies), and either the telomere primer pair or the Hbg primer pair, each primer at a final concentration of 200 nM. For each sample, quadruplicates of telomere and quadruplicates of Hbg reaction solutions were amplified in the same qPCR run, in the Rotor-Gene Q instrument operated with Q-series software version 2.0.2.4 (Qiagen). The qPCR conditions consisted of three steps with melt, beginning with 95°C incubation for 3 minutes, followed by 40 cycles of: 95°C for 20 sec, 56°C for 60 sec and 72°C for 20 sec. After PCR amplification, melting curves were generated to confirm the specificity of PCR products. A negative control (no DNA template) and a reference DNA sample for normalization between experiments were run in duplicates in each batch. This same reference DNA sample was used to generate standard curves for telomere and Hbg amplifications; efficiency was 90% and 92%, respectively. The mean cycle threshold (Ct) values for both telomere and Hbg at a fluorescence signal threshold of 0.3 were calculated from the three closest values of quadruplicate samples with exclusion of the fourth value when it fell outside two standard deviations (SD) from the mean [20, 21]. The intra-assay coefficient of variation (CV) of the Ct was 1.80% and 0.92% and the inter-assay CV was 3.59% and 2.50% for telomeres and Hbg respectively. The comparative Ct method was used for relative quantification of telomere length, using this formula: relative T/S ratio = 2^-ΔΔCt^ where ΔΔCt = (Ct _Telomere_−Ct _Hbg_) _sample_—(Ct _Telomere_−Ct _Hbg_) _reference DNA_ [22]. All assays were performed blinded to the study patients’ characteristics and clinical data.

### Statistical analysis

Telomere length, measured as a relative T/S ratio, was treated as a continuous variable, which is typically positively skewed. A Box-Cox transformation method was used to determine the suitable power transformation for the relative T/S ratio to obtain a normal distribution. General linear models (GLM, models fitted by least squares and weighted least squares using SAS Proc GLM) were conducted to evaluate the association between the square root-transformed relative T/S ratio and each of a set of pre-specified prognostic factors: age (years; quintiles), menopausal status, body mass index (BMI) (kg/m^2^; quintiles), smoking status (never, former, current), alcohol consumption (drinks per week; quintiles), physical activity (MET-hours of activity per week; quintiles), TNM stage (0, I, II, III), histological type (in-situ ductal, invasive ductal, invasive lobular, other), tumor grade (1, 2, 3), estrogen receptor (ER) status (negative, positive), progesterone receptor (PR) status (negative, positive) and human epidermal growth factor receptor 2 (HER2) status (negative, positive). For one variable (transportation-related physical activity), 77% of the values were zeros, which generated empty cells and less than five observations in all quintile categories; consequently, this variable was dichotomized (presence *vs* absence of transportation-related physical activity). To comply with statistical modeling assumptions, age- and menopausal status-adjusted associations between the square root-transformed relative T/S ratio and each factor were estimated. The same analyses were performed with stratification for menopausal status. The resulting adjusted estimates were back transformed to adjusted means of relative T/S ratio. Contrast statements were computed to generate tests for linear trends, using appropriate orthogonal polynomial coefficients for unequally spaced means in the GLM procedure. Spearman partial correlations of the relative T/S ratio and each of the above factors, while adjusting for age and menopausal status were computed. Inclusion of all the pre-specified factors in multivariable models and Spearman partial correlations did not change the observed associations, hence, age- and menopausal status- adjusted estimates are presented.

Given that our sample size was fixed at 162, the present study was powered to detect a significant correlation ≥0.20 and at least a 0.3 standardized difference with 80% power and a two-sided statistical significance of 5% [23]. All statistical analyses were performed with SAS software version 9.4.

## Results

Telomere length was estimated for all of the 162 Caucasian patients (mean ± SD of relative T/S ratio = 1.06 ± 0.63, median = 0.97, range 0.04 to 3.04). Characteristics of study participants are presented in Table 1. Patients were aged between 30 and 69 years (median = 52), and 50% were pre-menopausal. The majority had an invasive ductal carcinoma stage I or II and none had distant metastasis. More post-menopausal women were obese (28.4% with BMI ≥30 kg/m^2^) and former smokers (53.1%) compared to premenopausal women (16.1% with BMI ≥30 kg/m^2^; 35.8% former smokers). Pre-menopausal women were more active, with higher total (137.5 ± 47.2 *vs* 99.7 ± 48.6 MET-hours per week) and occupational (79.9 ± 37.6 *vs* 47.4 ± 47.3 MET-hours per week) physical activity. Post-menopausal women had more advanced disease with regard to stage (14.8% *vs* 5% stage III), more invasive lobular tumors (13.6% *vs* 4.9%), more hormonal negative tumors (12.4% *vs* 8.6% for ER, 23.5% *vs* 8.6% for PR), and slightly more HER2 positive tumors (12.3% *vs* 9.9%) compared to pre-menopausal women.

**Table 1:** Patient characteristics.

	All women	Pre-menopausal	Post-menopausal
	n = 162	n = 81	n = 81
Number (%)			
**Age** (mean ± SD, years)	52.6 ± 7.9	46.8 ± 5.8	58.3 ± 4.9
**BMI** (mean ± SD, kg/m^2^)	27.0 ± 5.6	26.3 ± 5.6	27.7 ± 5.5
<25	67 (41.4)	39 (48.2)	28 (34.6)
25-<30	59 (36.4)	29 (35.8)	30 (37.0)
≥30	36 (22.2)	13 (16.1)	23 (28.4)
**Smoking status**			
Never	69 (42.6)	41 (50.6)	28 (34.6)
Former	72 (44.4)	29 (35.8)	43 (53.1)
Current	21 (13.0)	11 (13.6)	10 (12.4)
**Alcohol consumption** (mean ± SD, drink per week)	4.3 ± 4.6	4.5 ± 4.2	4.1 ± 5.0
**Physical activity** (mean ± SD, MET-hours per week)			
Total	118.6 ± 51.4	137.5 ± 47.2	99.7 ± 48.6
Occupational	63.6 ± 45.6	79.9 ± 37.6	47.4 ± 47.3
Transportation-related	0.7 ± 2.0	0.8 ± 2.3	0.7 ± 1.7
Household	36.0 ± 23.3	36.2 ± 24.0	35.7 ± 22.6
Recreational	18.3 ± 17.0	20.6 ± 19.1	16.0 ± 14.3
**Stage**			
0	16 (9.9)	9 (11.1)	7 (8.6)
I	64 (39.5)	33 (40.7)	31 (38.3)
II	66 (40.7)	35 (43.2)	31 (38.3)
III	16 (9.9)	4 (5.0)	12 (14.8)
**Histological type**			
Ductal, in-situ	16 (9.9)	9 (11.1)	7 (8.6)
Ductal, invasive	121 (74.7)	66 (81.5)	55 (67.9)
Lobular, invasive	15 (9.3)	4 (4.9)	11 (13.6)
Others*	10 (6.1)	2 (2.5)	8 (9.9)
**Tumor grade**			
Non-assessable	26 (16.0)	11 (13.6)	15 (18.5)
1	29 (17.9)	15 (18.5)	14 (17.3)
2	68 (42.0)	36 (44.4)	32 (39.5)
3	39 (24.1)	19 (23.5)	20 (24.7)
**ER status**			
Positive	145 (89.5)	74 (91.4)	71 (87.6)
Negative	17 (10.5)	7 (8.6)	10 (12.4)
**PR status**			
Positive	136 (84.0)	74 (91.4)	62 (76.5)
Negative	26 (16.0)	7 (8.6)	19 (23.5)
**HER2 status**			
Not evaluated	28 (17.3)	11 (13.6)	17 (21.0)
Positive	18 (11.1)	8 (9.9)	10 (12.3)
Negative	116 (71.6)	62 (76.5)	54 (66.7)

SD: Standard deviation; BMI: Body mass index; MET-hours: metabolic equivalent hours of activity; ER: Estrogen receptor; PR: Progesterone receptor; HER2: Human epidermal growth factor receptor 2

*: includes mucinous, tubular, adenoid cystic and metaplastic carcinomas

Spearman correlations coefficients and results from the GLM models of the associations of telomere length and traditional prognostic factors are presented in Table 2. Telomere length was not associated with age or with menopausal status. No association was observed for telomere length with stage, histological type, tumor grade, ER status, PR status and HER2 status, either before or after stratification according to menopausal status (Table 2).

**Table 2:** Associations of telomere length and traditional prognostic factors.

All women			Pre-menopausal		Post-menopausal	
	Mean (95% CI)	Adjusted* mean (95% CI)		Adjusted* mean (95% CI)		Adjusted* mean (95% CI)
**Age** (quintiles, years)						
30–47	1.16 (0.96, 1.38)	1.21 (0.95, 1.50)	30–42	1.05 (0.78, 1.36)	43–54	1.21 (0.94, 1.53)
48–51	0.92 (0.75, 1.12)	0.96 (0.75, 1.19)	43–47	1.26 (0.94, 1.63)	55–57	0.94 (0.70, 1.21)
52–55	1.15 (0.91, 1.43)	1.15 (0.91, 1.42)	48–50	0.99 (0.74, 1.28)	58–59	1.19 (0.90, 1.52)
56–59	1.09 (0.89, 1.31)	1.04 (0.81, 1.31)	51	0.78 (0.50, 1.15)	60–62	1.14 (0.83, 1.50)
60–69	0.99 (0.78, 1.22)	0.94 (0.70, 1.23)	52–57	1.15 (0.81, 1.56)	63–69	0.86 (0.62, 1.14)
*p-trend*	*0*.*475*	*0*.*365*		*0*.*633*		*0*.*180*
r_s_ (*p-value*)	-0.035 (*0*.*659*)	-0.079 (*0*.*321*)		-0.037 (*0*.*746*)		-0.150 (*0*.*182*)
**Menopausal status**						
Pre-menopausal	1.04 (0.91, 1.18)	0.97 (0.82, 1.14)				
Post-menopausal	1.07 (0.94, 1.21)	1.14 (0.97, 1.33)				
*p-value*	*0*.*777*	*0*.*227*				
r_s_ (*p-value*)	0.005 (*0*.*947*)	0.073 (*0*.*361*)				
**Stage**						
0	0.93 (0.67, 1.24)	0.93 (0.79, 1.24)		0.80 (0.48, 1.21)		1.13 (0.72, 1.64)
I	1.09 (0.94, 1.26)	1.09 (0.94, 1.25)		1.04 (0.83, 1.27)		1.15 (0.94, 1.38)
II	1.06 (0.91, 1.22)	1.06 (0.92, 1.22)		1.16 (0.94, 1.40)		0.96 (0.77, 1.17)
III	1.04 (0.76, 1.37)	1.03 (0.75, 1.37)		0.92 (0.43, 1.63)		1.07 (0.76, 1.44)
*p-trend*	*0*.*660*	*0*.*672*		*0*.*649*		*0*.*682*
r_s_ *(p-value)*	-0.001 (*0*.*994*)	-0.002 (*0*.*976*)		0.099 (*0*.*383*)		-0.102 (*0*.*370*)
**Histological type**						
Ductal, in-situ	0.93 (0.67, 1.24)	0.93 (0.67, 1.28)		0.80 (0.48, 1.21)		1.13 (0.72, 1.64)
Ductal, invasive	1.06 (0.95, 1.18)	1.06 (0.95, 1.18)		1.07 (0.92, 1.24)		1.04 (0.89, 1.20)
Lobular, invasive	1.03 (0.75, 1.37)	1.06 (0.77, 1.38)		1.48 (0.82, 2.37)		0.95 (0.64, 1.33)
Others*, invasive	1.24 (0.86, 1.70)	1.25 (0.86, 1.70)		1.00 (0.34, 2.10)		1.32 (0.90, 1.83)
*p-value*	*0*.*673*	*0*.*676*		*0*.*374*		*0*.*592*
r_s_ *(p-value)*	0.064 (*0*.*416*)	0.071 (*0*.*370*)		0.173 (*0*.*125*)		0.006 (*0*.*958*)
**Tumor grade**						
1	1.05 (0.84, 1.29)	1.04 (0.83, 1.28)		1.10 (0.78, 1.47)		0.98 (0.71, 1.31)
2	1.08 (0.94, 1.24)	1.09 (0.95, 1.25)		1.10 (0.89, 1.34)		1.09 (0.89, 1.30)
3	1.07 (0.88, 1.27)	1.06 (0.88, 1.27)		1.16 (0.87, 1.50)		0.97 (0.74, 1.24)
*p-trend*	*0*.*909*	*0*.*886*		*0*.*792*		*0*.*947*
r_s_ *(p-value)*	0.043 (*0*.*587*)	0.050 (*0*.*528*)		0.176 (*0*.*119*)		-0.080 (*0*.*480*)
**ER status**						
Positive	1.11 (0.82, 1.43)	1.05 (0.96, 1.16)		1.07 (0.92, 1.22)		1.04 (0.91, 1.19)
Negative	1.05 (0.95, 1.15)	1.07 (0.79, 1.40)		0.94 (0.54, 1.46)		1.17 (0.81, 1.60)
*p-value*	*0*.*732*	*0*.*905*		*0*.*611*		*0*.*540*
r_s_ *(p-value)*	-0.043 (*0*.*590*)	-0.033 (*0*.*681*)		0.047 (*0*.*680*)		-0.099 (*0*.*382*)
**PR status**						
Positive	1.05 (0.94, 1.15)	1.05 (0.95, 1.16)		1.07 (0.92, 1.22)		1.04 (0.90, 1.19)
Negative	1.12 (0.88, 1.38)	1.08 (0.85, 1.35)		0.92 (0.54, 1.46)		1.13 (0.87, 1.43)
*p-value*	*0*.*602*	*0*.*811*		*0*.*611*		*0*.*558*
r_s_ *(p-value)*	-0.044 (*0*.*578*)	-0.029 (*0*.*715*)		0.047 (*0*.*680*)		-0.079 (*0*.*488*)
**HER2 status**						
Positive	1.08 (0.81, 1.38)	1.07 (0.81, 1.38)		1.12 (0.95, 1.29)		1.03 (0.70, 1.17)
Negative	1.06 (0.95, 1.18)	1.06 (0.95, 1.18)		1.12 (0.71, 1.65)		1.01 (0.86, 1.43)
*p-value*	*0*.*938*	*0*.*947*		*0*.*979*		*0*.*914*
r_s_ *(p-value)*	0.031 (*0*.*695*)	0.043 (*0*.*593*)		0.188 (*0*.*094*)		-0.104 (*0*.*359*)

Adjusted means and p-trend values from the general linear models (GLM); * Adjusted for: age and menopausal status, when applicable; r_s_: Spearman correlation coefficient; ER: Estrogen receptor; PR: Progesterone receptor; HER2: Human epidermal growth factor receptor 2; *: includes mucinous, tubular, adenoid cystic and metaplastic carcinomas

**Fig 1:**
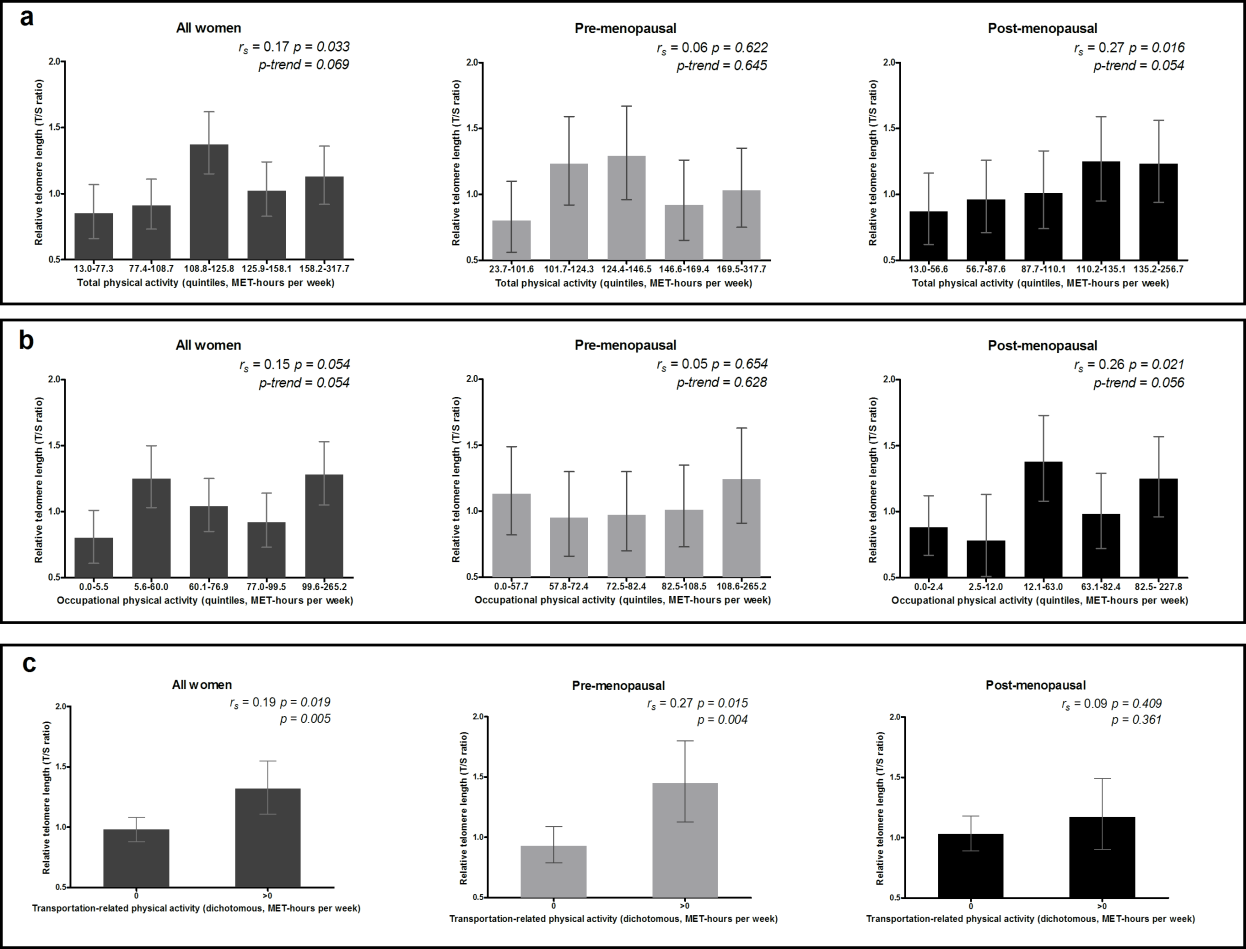
Association of telomere length with levels of total, occupational and transportation-related physical activity. Adjusted means and 95% confidence intervals of relative telomere length according to: (a) total physical activity (quintiles), (b) occupational physical activity (quintiles) and (c) transportation-related physical activity (dichotomous) for all, pre-menopausal and post-menopausal women, with adjustment for age (continuous) and menopausal status when applicable. a, b, c: MET-hours per week: metabolic equivalent hours of activity per week.

Spearman correlations coefficients and results from the GLM models of the associations of telomere length and lifestyle factors are presented in Table 3. Telomere length increased linearly with increasing levels of total physical activity (r_s_ = 0.17, *P = 0*.*033; P*_*trend*_ *= 0*.*069*), occupational physical activity (r_s_ = 0.15, *P = 0*.*054; P*_*trend*_ *= 0*.*054*) and transportation-related physical activity (r_s_ = 0.19, *P = 0*.*019; P = 0*.*005*) (Table 3 and Fig 1). When stratified by menopausal status (Table 3 and Fig 1), linear trends for increasing telomere length were observed for total physical activity (r_s_ = 0.27, *P = 0*.*016; P*_*trend*_ *= 0*.*054*) and occupational physical activity (r_s_ = 0.26, *P = 0*.*021; P*_*trend*_ *= 0*.*056*) in post-menopausal women, while in pre-menopausal women, TL was only associated with transportation-related physical activity (r_s_ = 0.27, *P = 0*.*015; P = 0*.*004*). No associations were observed for telomere length with recreational or household activities, or the other lifestyle factors considered, namely BMI, smoking status and alcohol consumption, either before or after stratification according to menopausal status (Table 3).

**Table 3:** Association of telomere length and lifestyle factors.

All women			Pre-menopausal		Post-menopausal	
	Mean (95% CI)	Adjusted* mean (95% CI)		Adjusted* mean (95% CI)		Adjusted* mean (95% CI)
**BMI** (quintiles, kg/m^2^)						
17.2–22.2	1.14 (0.93, 1.38)	1.13 (0.92, 1.37)	17.3–21.6	1.27 (0.94, 1.65)	17.2–22.8	0.95 (0.70, 1.25)
22.3–24.8	0.95 (0.76, 1.16)	0.95 (0.76, 1.16)	21.7–24.1	0.99 (0.70, 1.35)	22.9–25.5	1.33 (1.04, 1.67)
24.9–27.0	1.13 (0.91, 1.36)	1.13 (0.91, 1.36)	24.2–26.2	0.83 (0.58, 1.14)	25.6–27.4	1.06 (0.79, 1.38)
27.1–30.6	1.08 (0.88, 1.31)	1.10 (0.89, 1.33)	26.3–29.3	1.11 (0.79, 1.48)	27.5–32.1	0.90 (0.66, 1.19)
30.7–48.6	1.00 (0.80, 1.22)	0.98 (0.78, 1.20)	29.4–45.9	1.11 (0.81, 1.46)	32.2–48.6	1.04 (0.78, 1.34)
*p-trend*	*0*.*592*	*0*.*544*		*0*.*900*		*0*.*710*
r_s_ (*p-value*)	-0.040 (*0*.*614*)	-0.039 (*0*.*628*)		-0.039 (*0*.*732*)		-0.035 (*0*.*760*)
**Smoking status**						
Never	1.05 (0.91, 1.21)	1.07 (0.82, 1.36)		1.01 (0.82, 1.22)		1.12 (0.90, 1.36)
Former	1.06 (0.92, 1.21)	1.06 (0.92, 1.21)		1.06 (0.83, 1.32)		1.05 (0.88, 1.24)
Current	1.06 (0.81, 1.35)	1.05 (0.91, 1.20)		1.22 (0.84, 1.68)		0.92 (0.61, 1.30)
*p-trend*	*0*.*974*	*0*.*911*		*0*.*361*		*0*.*360*
r_s_ (*p-value*)	0.004 (*0*.*964*)	0.001 (*0*.*994*)		-0.074 (*0*.*513*)		0.098 (*0*.*389*)
**Alcohol consumption** (quintiles, drink/week)						
0–0.2	1.15 (0.89, 1.44)	1.13 (0.88, 1.43)	0.0–0.2	1.28 (0.93, 1.69)	0.0–0.1	1.09 (0.79, 1.43)
0.3–2.0	1.09 (0.92, 1.28)	1.09 (0.92, 1.27)	0.3–3.0	0.81 (0.60, 1.05)	0.2–1.0	1.18 (0.92, 1.48)
2.1–5.0	0.96 (0.77, 1.17)	0.94 (0.75, 1.16)	3.1–5.0	1.05 (0.74, 1.42)	1.1–4.0	1.08 (0.81, 1.41)
5.1–7.0	1.04 (0.84, 1.26)	1.05 (0.85, 1.27)	5.1–7.0	1.09 (0.80, 1.43)	4.1–7.0	0.98 (0.74, 1.25)
7.1–28.0	1.07 (0.83, 1.35)	1.11 (0.86, 1.39)	7.1–16.0	1.27 (0.89, 1.71)	7.1–28.0	0.93 (0.63, 1.30)
*p-trend*	*0*.*758*	*0*.*988*		*0*.*412*		*0*.*300*
r_s_ (*p-value*)	-0.070 (*0*.*377*)	-0.055 (*0*.*490*)		0.041 (*0*.*719*)		-0.127 (*0*.*260*)
**Physical activity** (quintiles, MET-hours per week)						
**Total**						
13.0–77.3	0.86 (0.69, 1.06)	0.85 (0.66, 1.07)	23.7–101.6	0.80 (0.56, 1.10)	13.0–56.6	0.87 (0.62, 1.16)
77.4–108.7	0.92 (0.74, 1.12)	0.91 (0.73, 1.11)	101.7–124.3	1.23 (0.92, 1.59)	56.7–87.6	0.96 (0.71, 1.26)
108.8–125.8	1.38 (1.15, 1.63)	1.37 (1.15, 1.62)	124.4–146.5	1.29 (0.96, 1.67)	87.7–110.1	1.01 (0.74, 1.33)
125.9–158.1	1.01 (0.82, 1.22)	1.02 (0.83, 1.24)	146.6–169.4	0.92 (0.65, 1.26)	110.2–135.1	1.25 (0.95, 1.59)
158.2–317.7	1.11 (0.91, 1.34)	1.13 (0.92, 1.36)	169.5–317.7	1.03 (0.75, 1.35)	135.2–256.7	1.23 (0.94, 1.56)
*p-trend*	*0*.*068*	*0*.*069*		*0*.*645*		*0*.*054*
r_s_ (*p-value*)	0.163 (*0*.*038*)	0.169 (*0*.*033*)		0.056 (*0*.*622*)		0.269 (*0*.*016*)
**Occupational**						
0.0–5.5	0.82 (0.65, 1.01)	0.80 (0.61, 1.01)	0.0–57.7	1.13 (0.82, 1.49)	0.0–2.4	0.88 (0.67, 1.12)
5.6–60.0	1.27 (1.05, 1.51)	1.25 (1.03, 1.50)	57.8–72.4	0.95 (0.66, 1.30)	2.5–12.0	0.78 (0.51, 1.13)
60.1–76.9	1.02 (0.84, 1.23)	1.04 (0.85, 1.25)	72.5–82.4	0.97 (0.70, 1.30)	12.1–63.0	1.38 (1.08, 1.73)
77.0–99.5	0.91 (0.72, 1.12)	0.92 (0.73, 1.14)	82.5–108.5	1.01 (0.73, 1.35)	63.1–82.4	0.98 (0.72, 1.29)
99.6–265.2	1.27 (1.05, 1.51)	1.28 (1.05, 1.53)	108.6–265.2	1.24 (0.91, 1.63)	82.5–227.8	1.25 (0.96, 1.57)
*p-trend*	*0*.*056*	*0*.*054*		*0*.*628*		*0*.*056*
r_s_ (*p-value*)	0.151 (*0*.*056*)	0.153 (*0*.*054*)		0.051 (*0*.*654*)		0.258 (*0*.*021*)
**Physical activity** (quintiles, MET-hours per week)						
**Transportation-related**^**†**^						
0	0.98 (0.88, 1.08)	0.98 (0.88, 1.08)		0.93 (0.79, 1.09)		1.03 (0.89, 1.18)
>0	1.33 (1.11, 1.56)	1.32 (1.11, 1.55)		1.45 (1.13, 1.80)		1.17 (0.90, 1.49)
*p-value*	*0*.*004*	*0*.*005*		*0*.*004*		*0*.*361*
r_s_ (*p-value*)	0.192 (*0*.*014*)	0.186 (*0*.*019*)		0.271 (*0*.*015*)		0.094 (*0*.*409*)
**Household**						
7.1–17.5	1.06 (0.86, 1.29)	1.07 (0.87, 1.30)	7.1–17.5	0.96 (0.70, 1.28)	8.7–18.2	1.19 (0.90, 1.51)
17.6–22.6	0.99 (0.79, 1.21)	0.99 (0.79, 1.22)	17.6–21.0	0.94 (0.66, 1.28)	18.3–24.2	1.03 (0.77, 1.34)
22.7–35.8	1.06 (0.86, 1.28)	1.05 (0.85, 1.27)	21.1–36.7	1.32 (0.98, 1.71)	24.3–35.2	0.81 (0.57, 1.09)
35.9–53.6	1.20 (0.98, 1.44)	1.19 (0.97, 1.43)	36.8–52.5	1.15 (0.83, 1.54)	35.3–53.6	1.21 (0.92, 1.55)
53.7–148.6	0.99 (0.79, 1.21)	0.99 (0.79, 1.21)	52.6–113.9	0.94 (0.67, 1.25)	53.7–148.6	1.07 (0.80, 1.38)
*p-trend*	*0*.*913*	*0*.*882*		*0*.*824*		*0*.*890*
r_s_ (*p-value*)	0.040 (*0*.*610*)	0.038 (*0*.*638*)		0.084 (*0*.*460*)		-0.009 (*0*.*938*)
**Recreational**						
0.0–3.8	0.98 (0.79, 1.20)	0.99 (0.79, 1.10)	0.0–5.2	1.17 (0.85, 1.55)	0.0–3.3	0.90 (0.65, 1.20)
3.9–9.4	1.11 (0.90, 1.34)	1.10 (0.89, 1.34)	5.3–11.0	1.06 (0.77, 1.41)	3.4–8.8	1.15 (0.87, 1.47)
9.5–19.0	1.14 (0.93, 1.37)	1.14 (0.94, 1.37)	11.1–19.5	0.94 (0.65, 1.28)	8.9–14.5	1.14 (0.84, 1.48)
19.1–27.9	0.95 (0.75, 1.17)	0.95 (0.76, 1.17)	19.6–34.6	1.03 (0.75, 1.37)	14.6–27.4	1.08 (0.81, 1.39)
28.0–104.8	1.10 (0.89, 1.33)	1.09 (0.89, 1.32)	34.7–104.8	1.09 (0.78, 1.45)	27.5–61.9	1.04 (0.77, 1.36)
*p-trend*	*0*.*810*	*0*.*875*		*0*.*896*		*0*.*830*
r_s_ (*p-value*)	0.023 (*0*.*775*)	0.020 (*0*.*806*)		0.019 (*0*.*869*)		0.042 (*0*.*714*)

Adjusted means and p-trend values from the general linear models (GLM)

* Adjusted for: age and menopausal status, when applicable

^†^: Dichotomous categories (0: n = 125; >0: n = 37); r_s_: Spearman correlation coefficient; BMI: Body mass index; MET-hours: Metabolic equivalent hours of activity

## Discussion

The present study aimed at assessing the association of telomere length with traditional and potential prognostic factors. The findings suggest that peripheral white blood cell telomeres are longer in more active breast cancer patients, especially for transportation-related physical activity among pre-menopausal patients, and for total and occupational physical activity among post-menopausal patients. Neither age nor menopausal status, nor tumor prognostic factors nor certain modifiable factors were associated with peripheral white blood cell telomere length.

Although modest associations of physical activity with telomere length have been reported in healthy individuals (an increase of 0.07-SD of relative telomere length in moderately or highly active women *vs* least active women) [9] and breast cancer patients (β = −0.22, 95% confidence interval (CI): -0.41 to -0.03, n = 392 post-menopausal women) [24], the present study is the first to reveal associations of different types of physical activity with telomere length in pre- and post-menopausal breast cancer patients. Mean MET-hours per week of total physical activity in our population was higher than total energy expenditure recommended for healthy adults to achieve health benefits while mean MET-hours per week of transportation-related physical activity was relatively very low. In fact, based on a systematic review of 254 studies there is a dose-response relationship between increased physical activity and health benefits [25]. To achieve health benefits, healthy adults should accumulate at least 150 minutes of moderate- to vigorous-intensity aerobic physical activity per week, which corresponds to an energy expenditure comprised between 500 and 1,000 MET-minutes per week (8.33 and 16.67 MET-hours per week) [26, 27]. Our findings suggest a similar dose-response relationship between physical activity and telomere length, but not for all physical activity domains, and depending on the menopausal status. Low intensity, but probably regularly performed, physical activity seems to be associated with longer peripheral blood cell telomere length. Hence, breast cancer patients, for whom the recommendation is to engage in regular physical activity at least 150 minutes per week [28], but among whom moderate to vigorous exercise may be difficult to achieve, may benefit from regular low-intensity physical activity.

Even though older age was found to be related to shorter telomere length in healthy women, with statistically significant correlation coefficients varying from −0.09 (p-value <0.001, n = 7813, of whom 80% were post-menopausal women) [9] to –0.23 (p-value <0.04, n = 58 premenopausal women) [8], it is probably not the only determinant of telomere shortening in breast cancer patients. In fact, only one out of five studies reported a statistically significant adjusted association of older age with shorter peripheral white blood cell telomere length [15], when comparing patients less than 55 years old to those older than 65 (β = −0.26, p-value = 0.02, n = 392 post-menopausal women) [24]. The same observation was made for other modifiable factors (BMI, smoking, alcohol consumption) which were found to be associated with shorter telomere length in healthy women [7, 10], but not in breast cancer patients [15]. Only two studies have assessed the associations of tumor prognostic factors with peripheral blood cell relative telomere length, and one reported no association with ER status after adjustment for age [15, 29]. Moreover, longitudinal cohort studies suggest that telomere length might be an independent prognostic factor [15].

The strengths of the present study include the recruitment of a consecutive series of women presenting with breast cancer and the high participation percentage among eligible women (73%), which minimizes the risk of selection bias. Even though pre-menopausal women constituted half of our population, the distributions of study patients by age category–with 70.4% aged older than 50 years–and tumor characteristics—74.7% had ductal invasive carcinomas and 89.5%, ER-positive tumors—were very similar to those of the breast cancer population [30]. Additionally, telomere length was estimated for all patients who provided blood samples and all participants were included in statistical analyses. Data collection at time of surgery using standardized measures and questionnaires, the use of an appropriate DNA extraction method and the assessment of telomere length with an appropriate and highly reproducible method ensured the quality of the data, and prevented selection bias resulting from missing values and measurement bias. All the participants were approached for information about risk factors when they were not yet aware of their disease severity and stage (an average of 24 days after surgery) or their telomere length, which prevents recall bias. All the laboratory assays were performed blinded to study patient characteristics and clinical data, which prevent bias from differential misclassification. Thus, if a measurement error had occurred, it would result in non-differential misclassification, and would have underestimated the true associations between telomere length and the factors studied. The estimates presented were all adjusted for age and menopausal status, and were not different from those adjusted for all the pre-specified known and potential prognostic factors. However, residual confounding from unknown factors, a common concern in observational studies, may still exist.

The limitations include the cross-sectional design that precludes causal inferences. However, it seems very likely that the collected information about risk factors refers to exposure before blood collection, especially physical activity, for which questions were derived from the Past Year Total Physical Activity Questionnaire [17]. Even though peripheral white blood cell telomere length seems to be a dynamic feature [14], it is likely that factors affecting telomere length have a long latency period, as observed with chemotherapy, which seems to induce an initial telomere attrition 3–6 months after treatment that takes 1–5 years to recover [31, 32]. The relatively small sample size could also have been a limitation of the present study. However, the strength of the relationships between telomere length and the studied factors, as reflected by the size of correlation coefficients regardless of statistical significance, were as high as those observed in larger studies [8, 9].

Finally, given that white blood cells are involved in anticancer immune responses, they are likely to be linked with breast cancer prognosis. A longitudinal analysis of the association of telomere length at the time of diagnosis, as a surrogate for both innate adaptive abilities and cumulative exposures to modifiable environmental factors, with survival is still needed to demonstrate the significance of telomere length as an independent prognostic marker.

## Conclusions

Longer peripheral blood cell telomeres seem to be associated with higher levels of physical activity in breast cancer patients, especially for physical activity related to occupation and transportation. Telomere length was not associated with any of the other known or potential prognostic factors. These findings suggest that even regular low-intensity physical activity could be effectively recommended to breast cancer patients, and may contribute to the control of cancer along with conventional therapies.
